# A community approach for pathogens and their arthropod vectors (ticks and fleas) in cats of sub-Saharan Africa

**DOI:** 10.1186/s13071-022-05436-y

**Published:** 2022-09-09

**Authors:** Maxime Madder, Michael Day, Bettina Schunack, Josephus Fourie, Michel Labuschange, Wouter van der Westhuizen, Sherry Johnson, Samuel Maina Githigia, Foluke Adedayo Akande, Jahashi Saidi Nzalawahe, Dickson Stuart Tayebwa, Ortwin Aschenborn, Mary Marcondes, Dieter Heylen

**Affiliations:** 1 Clinglobal, Tamarin, Mauritius; 2 School of Veterinary and Life Sciences, Murdoch University, Murdoch, WA Australia; 3 Elanco Animal Health, Leverkusen, Germany; 4 Clinvet, Waverly, USA; 5 Clinvet, Bloemfontein, South Africa; 6 Clinomics, Bloemfontein, South Africa; 7 School of Veterinary Medicine, CBAS, University of Ghana, Accra, Ghana; 8 Department of Veterinary Pathology, Microbiology & Parasitology, University of Nairobi, Nairobi, Kenya; 9 Department of Veterinary Parasitology and Entomology, College of Veterinary Medicine, Federal University of Agriculture, Abeokuta, Nigeria; 10 Sokoine University of Agriculture, Morogoro, Tanzania; 11 Research Center for Tropical Diseases and Vector Control, College of Veterinary Medicine, Animal Resources and Biosecurity, Makerere University, Kampala, Uganda; 12 School of Veterinary Medicine, University of Namibia, Ondekaremba, Namibia; 13 São Paulo State University, São Paulo, Brazil; 14 Eco-Epidemiology Group, Department of Biomedical Sciences, Institute of Tropical Medicine, Antwerp, Belgium; 15 Interuniversity Institute for Biostatistics and Statistical Bioinformatics, Hasselt University, Diepenbeek, Belgium; 16 Department of Ecology and Evolutionary Biology, Princeton University, Princeton, NJ USA

**Keywords:** Cat, Sub-Saharan Africa, Ticks, Fleas, Vector-borne pathogens, *Ixodes*, *Haemaphysalis*, *Rhipicephalus*, *Amblyomma*, *Ctenocephalides*

## Abstract

**Background:**

Arthropod-borne pathogens and their vectors are present throughout Africa. They have been well studied in livestock of sub-Saharan Africa, but poorly studied in companion animals. Given their socioeconomic importance, the African Small Companion Animal Network (AFSCAN), as part of the WSAVA Foundation, initiated a standardized multi-country surveillance study.

**Methods:**

In six countries (Ghana, Kenya, Nigeria, Tanzania, Uganda, and Namibia) in both rural and urban settings, 160 infested cats were sampled to assess their ectoparasite community (ticks and fleas), as well as the micro-parasite prevalence within those ectoparasites (60 and 118 pools of ticks and fleas, respectively) and blood (276 cats, including 116 non-infested).

**Results:**

Almost two thirds of all infested cats originated from Tanzania and Kenya. Despite the large macro-geographical variation, no consistent difference was found in ectoparasite diversity and numbers between East and West Africa. Far more flea-infested than tick-infested cats were found. The most dominant ectoparasite was *Ctenocephalides felis*. Among the ticks, the exophilic *Haemaphysalis* spp. were the commonest, including species that are not typically linked with companion animals (*Haemaphysalis spinulosa* and *Haemaphysalis elliptica*). The most prevalent pathogens found in the blood and fleas were *Bartonella henselae* and *Mycoplasma haemofelis*. In the ticks, the dog-associated *Hepatozoon canis* was most commonly found. A high degree of co-parasitism was found in all countries and habitats.

**Conclusions:**

Our continent-wide standardized field study highlights the cat’s potential to serve as a reservoir of pathogens that can be transmitted to humans or livestock, especially when cats are expected to become more commonly kept in African villages and towns.

**Graphical Abstract:**

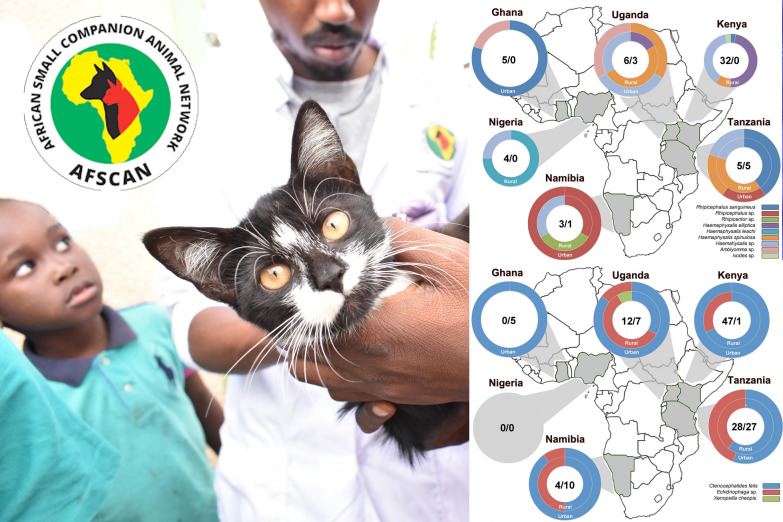

**Supplementary Information:**

The online version contains supplementary material available at 10.1186/s13071-022-05436-y.

## Background

The role of domestic carnivores like cats and dogs, as reservoirs of zoonotic and carnivore-specific pathogens, has been scarcely studied in low- and middle-income countries (LMICs), especially on the African continent, and hardly any recent data are available from studies set up in a uniform way with standardized diagnostic methods, making it difficult to compare pathogen populations across countries and regions. Particularly, cats are underappreciated in the scientific community [1] although they live in close relationship with their owners and other pets and livestock, making them a potential risk as reservoirs.

In a recent publication, pathogens and arthropod vectors from dogs were identified in sub-Saharan Africa [2]. As a follow-up study of the same project, the focus was put on another hypercarnivore, the domestic cat. Cats are considered keynote predatory species at the top of the food chain in diverse ecological niches, being it wild, rural, or urban [3]. The first domestic cats had limited utility and initiated their domestication among the earliest agricultural Neolithic settlements in the Near East [1]. Thereafter they became more adapted to humans although they were rather tolerated by humans and not domesticated as dogs were.

Nevertheless, it is important to determine the increased risk of human exposure to cat-associated parasites and pathogens in LMICs in the African continent as well, as economic growth favors pet adoption.

Ectoparasite and vector-borne pathogen abundances depend in a multidimensional way on the occurrence of suitable hosts in the ectoparasite habitat. The establishment and implementation of effective measures to control (zoonotic) diseases requires the understanding of the pathogen and reservoir hosts in a given geographical area [3]. Given the abovementioned importance of small companion animals, a multi-country surveillance study on ectoparasite (flea and tick) communities and the pathogens they transmit was initiated in sub-Saharan Africa. The World Small Animal Veterinary Association (WSAVA) and the African Small Companion Animal Network **(**AFSCAN), which focuses on enhancing companion animal veterinary care across Africa through the creation of a sustainable veterinary network for Africa, support this study, which attempts to identify the ectoparasites and vector-borne pathogens of cats in rural and urban areas in six African countries: Ghana, Kenya, Nigeria, Tanzania, Uganda, and Namibia. Based on the biological samples (ectoparasites, blood, and sera) we considered the following points: (1) to which degree do communities of ectoparasites and the pathogens they transmit vary macro-geographically, and (2) is there a difference in these communities from urbanized and rural areas.

## Methods

### Study design and site

**Fig. 1:**
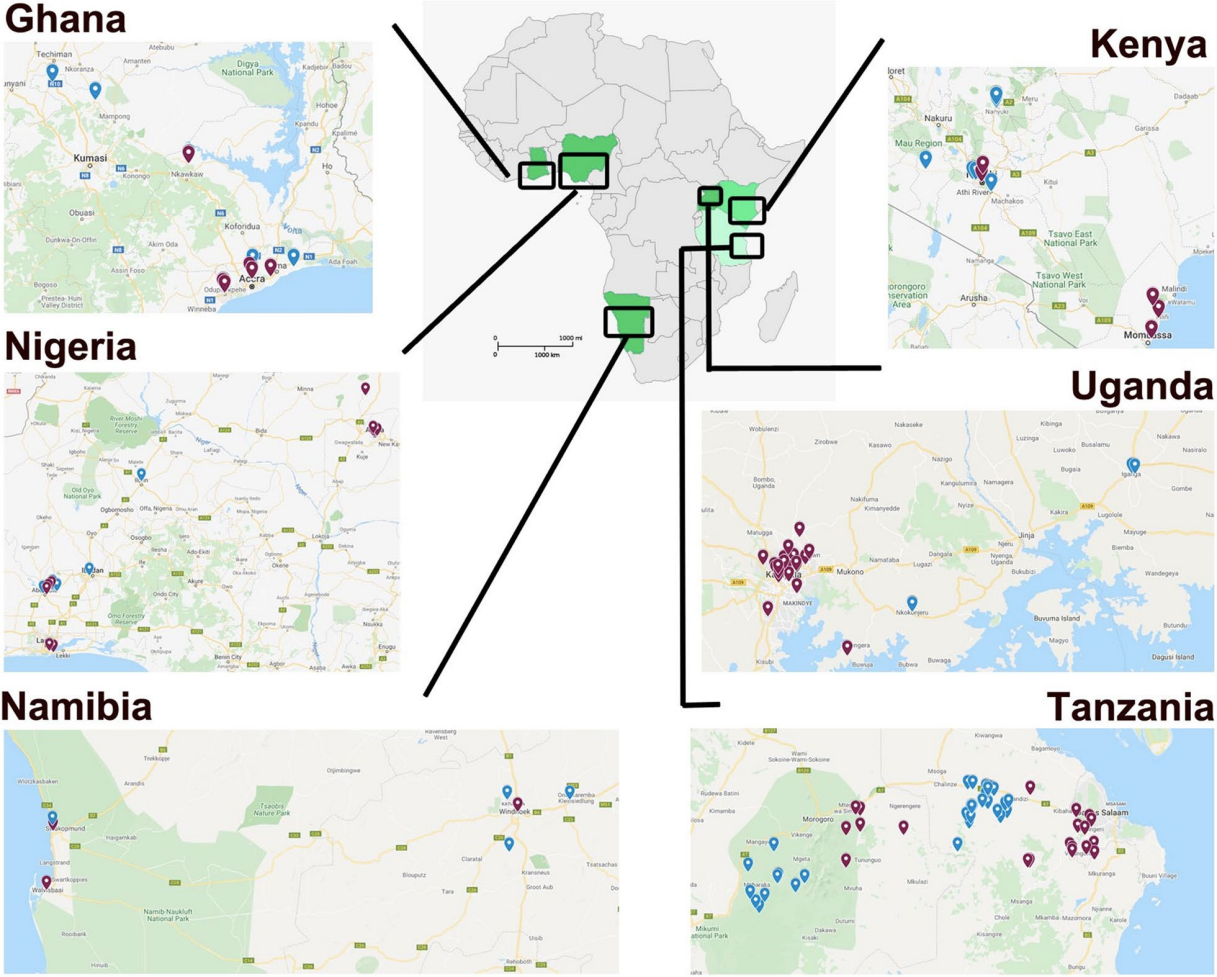
Overview of the sampling locations in the six African countries (Ghana, Kenya, Nigeria, Tanzania, Uganda, and Namibia). Location points in blue and red indicate rural and urban habitats, respectively

This is a multi-site field study to determine the present community of cat (*Felis catus*) ectoparasite species (ticks and fleas) and vector-borne pathogens. Preferably 50 ectoparasite-infested cats per country (Ghana, Kenya, Nigeria, Tanzania, Uganda, and Namibia) were sampled in both urban and rural areas (Fig. 1). Urban areas are described as cities with a high population (> 20,000 inhabitants) with extensive housing infrastructure (mainly offices and markets), a well-developed transportation network, and access to municipal water, contemporary housing, and electricity. Rural areas are defined as having a sparse population. The housing and infrastructure are poor; municipal water is scarce, with no electricity and hardly any access to public transport. The focus is on agricultural activities and cats hardly receive veterinary care. Cats of all countries were sampled and treated during the rainy season, except for Namibia (see Additional file 1: Fig. S1 for the timing of sampling).

In Ghana, the two regions that were sampled are the Greater Accra region (GAR) in the south of the country, and in the Ashanti region, Akumadan. In the GAR, samples were retrieved from private veterinary clinics in Accra (East Legon, Haatso-Atomic, Dome, Madina) and Tema metropolis. In Akumadan, the areas from where the cats originated were Nkwakwaa, Asempanaye, Asuosuo, and Afrancho. These are defined as rural and agricultural zones with moist semi-deciduous forests and thick vegetation cover with undergrowth. In Kenya, cats were sampled in Nairobi (the capital city) and Mombasa (a coastal urban town). These animals were well maintained and were provided housing, decent welfare, and veterinary care. In Narok, defined as a rural area and pastoral area, cats lived among livestock with likely contact with wild animals. In Uganda, the two urban areas were focused within the capital Kampala and Wakiso district in central Uganda, whereas the two rural areas were in Mbarara and Iganga municipalities which are located in the western and eastern regions of Uganda, respectively. Most of the cats in Wakiso and Kampala were well groomed, well fed, well treated, and confined in fenced houses although they could roam occasionally. On the contrary, cats in the rural areas which were kept particularly to keep vermin (mice and rats) at bay roamed freely, interacting with other pets and with livestock and wildlife. In Nigeria, the samples were taken from two urban and two rural settings in each of the North Central and Southwest geopolitical divisions of Nigeria. In the rural areas, farmers hardly had access to facilities for their use. In Tanzania, the region of Morogoro was selected as an urban area, and the region between Morogoro and Dar es Salaam, the capital, as a rural area. In Namibia, the area between Walvisbaai and Hettiesbaai was taken for both urban and rural areas, with urban sites more situated in the center of the cities and rural more inland. This area is located in the eastern part of the country, bordering the Atlantic Ocean.

To obtain the support of owners to commit animals for sampling, free rabies vaccination to companion animals and free ectoparasite preventive treatment for both cats and dogs were offered. During the field study, the owners’ consent was obtained and field assessment also involved obtaining details on animal sampled, physical examinations, scoring of tick-predilection sites, and sampling of ectoparasites and blood. Laboratory assessments included analyses of field samples related to ectoparasite species (ticks, fleas) and vector-borne parasite identification. Sample equipment, data forms, and IDEXX tests (see below) were made available to the sampling teams. The molecular analyses of blood samples spotted on FTA cards, ectoparasite species, and vector-borne pathogens, were performed centrally in a single lab (Clinomics, Bloemfontein, South Africa). Interpretation of the IDEXX kits was performed locally.

### Inclusion criteria

In urban areas, a link with a veterinary practice was established by each investigator. Samples were taken from privately owned cats visiting the veterinarian. In rural areas, where most cats were likely free-roaming and/or community-owned cats, sampling was performed based on the rabies vaccination/ectoparasite control provision for dogs, where cats were also presented. No sampling was performed at animal shelters. There were also no restrictions on breed or age. For each enrolled cat, age, sex, weight, body condition score (5-point scale: very thin, underweight, ideal, overweight, obese), housing (free-roaming, yard, indoor), parasiticide treatment (ectoparasiticide and deworming drugs), and presence of other cats or dogs were recorded using a standardized data capture form (see Additional file 2: capture form).

### Ectoparasite burden assessment and collection

For each cat, seven different body areas were systematically screened for ticks. For each body area, a burden score was assigned (0: absent, 1–2: one and two ticks respectively, > 3: three or more ticks). A cumulative number of ticks per individual cat was created (the scores of all body areas were summed) and for statistical analyses, three categories were created for the "No" (cumulative number: 0), "Moderate infestation" (0–2 ticks) and "High infestation" (> 2). Secondly, for fleas, the whole body was screened without recording details of the collection site. An estimate for the entire animal was obtained as follows: "No" (0 fleas), "Light infestation" (1–10 fleas), "Moderate infestation" (11–50 fleas), and "Severe infestation" (> 50 fleas). Up to 14 ticks were collected from each animal and placed into a plastic tube with a screw cap, containing 70% ethanol. All fleas were collected from the cat and placed together with the ticks into the same collection container (i.e., one container per animal).

### Blood collection and processing

A syringe and appropriate needle were used to collect whole blood samples. Blood was spotted on an FTA card (Whatman^®^) on which blood was preserved for DNA analyses. IDEXX 4Dx Plus Test kits were used to screen the serum for *Ehrlichia canis/Ehrlichia ewingii*, *Anaplasma phagocytophilum/Ehrlichia platys*, *Borrelia burgdorferi*, and *Dirofilaria immitis* following the manufacturer’s manual. For the serum preparation, plain collection tubes were centrifuged after the blood had clotted. The remaining serum was frozen at − 20 °C in plastic screw-cap tubes and stored for future research.

### Vector-borne pathogen identification

FTA card technology was used to ship blood samples. Cards were punched (3 × 5 mm diameter punches) for DNA isolation. Per cat, five ticks (or < 5 if fewer ticks were collected) and five fleas (or < 5 if fewer fleas were found) were randomly sampled from the container for molecular identification using multiplex polymerase chain reaction (PCR). Ticks and fleas from each individual cat were pooled separately for DNA isolation. These samples were homogenized by bead-bashing before processing using the MagMAX™ DNA Multi-Sample Ultra Kit (Thermo Fisher Scientific) according to the manufacturer’s specifications and eluted with 100 µl elution buffer.

Blood samples, ticks, and fleas were then screened for the presence of the most common tick- and flea-borne pathogens using PCR techniques: *Babesia rossi*, *Babesia canis*, *Babesia felis*, *Bartonella henselae*, *E. canis*, *Ehrlichia chaffeensis*, *Anaplasma platys*, *A. phagocytophilum*, *Rickettsia conorii*, *Rickettsia africae*, *Rickettsia felis*, *Coxiella burnetti*, *Hepatozoon canis*, and *Mycoplasma haemofelis*. Blood samples were also screened for a mosquito-borne (*D. immitis*) pathogen*.*

The isolated DNA (5 µl) served as template in 15 µl hydrolysis probe-based multiplex quantitative real-time PCR (qPCR) assay reactions using Luna^®^ Universal Probe qPCR Master Mix (New England Biolabs) to detect the target of interest according to the host (feline blood) and sample type (ticks, fleas). A universal thermal cycling program was used, which entailed a polymerase activation step at 95 °C for 3 min, followed by 45 cycles consisting of 15 s at 95 °C for denaturation and 30 s at 60 °C for elongation for all multiplexes excluding the *Dipylidium caninum* complex which had an extended elongation time of 60 s due to the longer expected amplicon size. The targets and their respective DNA templates are indicated in Table 1 and different multiplex reactions are in Additional file 1: Table S1.

**Table 1:** Overview of the targets and their respective DNA templates used in multiplex qPCR assay screening

Target	Feline blood	Tick	Flea	Limit of detection (copies/PCR)	References
*Babesia rossi*		X		5	[16]
*Babesia canis*		X		5	[16]
*Ehrlichia canis*		X		5	[17]
*Ehrlichia chaffeensis*		X		5	[18]
*Anaplasma platys*		X		16	[19]
*Anaplasma phagocytophilum*		X		9	[20]
*Rickettsia conorii*		X		8	[21]
*Rickettsia africae*		X		8	[21]
*Rickettsia felis*	X		X		[22]
*Coxiella burnetti*		X		8	[23]
*Hepatozoon canis*		X		5	In-house
*Dirofilaria immitis*		X		8	[24]^a^
*Bartonella henselae*	X		X	5	[25]
*Mycoplasma haemofelis*	X		X	16	[26]
*Babesia felis*	X	X		8	In-house^a^
*Dipylidium caninum*			X	8	[27]

^a^Primer sequences from the reference were used in conjunction with a hydrolysis probe, which was designed in-house (Clinomics, Bloemfontein, South Africa)

A first-round screening was performed on all extracted DNA samples using a positive extraction control to assess DNA isolation and an internal amplification control to assess template-derived inhibition of the PCR. For those samples where neither the internal amplification control nor any other targets were detected, they were diluted 1:1 using 10% Chelex (Bio-Rad laboratories). The results were analyzed using QuantStudio™ Real-Time PCR Software to identify samples with detectable levels of target DNA. All qPCR runs included a DNA-negative extraction control, a host-negative control indicating that the assays did not detect host DNA, a no-template control, and a positive control.

The design of the primers and probes (*H. canis*, *D. immitis*, and *B. felis*) was performed using Geneious (http://www.geneious.com/) and validated in silico using sequence data available on GenBank (Table 2).

**Table 2:** Overview of primers and probes used for the in-house qPCR screening of three pathogenic agents

Target	In-house forward primer	In-house reverse primer	In-house hydrolysis probe
*Hepatozoon canis*	GGCAGTGACGGTTAACGGGGG	GCACCAGACTTGCCCTCCAATTG	VIC-CCGGAGAGGGAGCCTGAGAAACGG-QSY
*Dirofilaria immitis*			Cy5-CTTTGGAATATGTGTTTTTTTGGAGAGCCCTC-BHQ3
*Babesia felis*	AAGAAGCTCGTAGTTGAATTTCTGCC	GAGAAGCCGAAGCAACACAAATCCAG	Cy5-TGCGTTTTCCGACTGGCTTGGCA-BHQ3

For all PCRs, the final forward and reverse primer concentrations were 400 nM. The final probe concentration was 200 nM

### Vector identification

Ectoparasites were only identified molecularly. PCR was performed using Q5^®^ Hot Start High-Fidelity 2X Master Mix in 10 µl reactions containing 2 µl of tick/flea isolated DNA using primers 5′-AAAGATGACCAAACTTGATCATTTAGAGG-3 and 5′-TCGATGAAGAACGCAGCCAGCT-3′ at a final concentration of 500 nM each, which amplifies the internal transcribed spacer 1 (ITS1) region of the ticks and fleas. Thermal cycling involved a polymerase activation step at 98 °C for 2 min followed by 30 cycles consisting of the following: 98 °C for 10 s, 65 °C for 20 s, and 72 °C for 75 s, followed by a final extension step at 72 °C for 5 min. The PCR products were sequenced and analyzed using the Basic Local Alignment Search Tool (BLAST) for identification.

For those ticks, which could not be identified using this sequenced region, primers which amplified the 16S ribosomal mitochondrial DNA (mtDNA) of the ticks were used [4]. The reactions were executed using Platinum™ SuperFi II PCR Master Mix in 10 µl reactions containing 2 µl of tick-isolated DNA with the primers at 500 nM final concentration each. Thermal cycling entailed a polymerase activation step at 98 °C for 2 min followed by 30 cycles consisting of the following: 98 °C for 10 s, 60 °C for 45 s, and 72 °C for 30 s, followed by a final extension step at 72 °C for 5 min.

The newly generated sequences were submitted to GenBank under accession numbers OP143941-OP143962 for the tick and flea species identified (*Ctenocephalis felis*, *R. sanguineus*, *Haemaphysalis leachi*, *H. spinulosa*, *Rhipicentor nuttalli*).

### Statistical analysis

The proportions of infested cats per ectoparasite taxon and their infestation intensities were compared between countries (Ghana, Kenya, Nigeria, Tanzania, Uganda, and Namibia), and urbanization level (urban vs. rural), as well as the proportion of pathogen-infected ectoparasite batches. We highlight that for each country, urban and rural locations are different (see descriptions above), meaning that a generalized continent-wide comparison of "urban versus rural" has little epidemiological relevance. For this purpose, generalized estimation equation models (GEE) were fitted to the data [5], which take into account the statistical dependence of observations in the same areas. The residuals for burden categories and pathogen proportions were assumed to follow a binomial distribution (logit-link, in ordinal and logistic regression, respectively). For the statistical comparison of the parasite community, Fisher’s exact tests were executed whereby the species distribution (in the population of parasitized individuals) was compared between habitat types (urban vs. rural) and countries. In addition, the Shannon diversity index was computed [6]. All prevalence estimates are reported as mean ± standard error. All data management and statistical analyses were done in SAS v 9.3 (SAS Institute, Cary, NC, USA).

## Results

### Ectoparasites

Of all infested cats examined (*n* = 160), 41.1% had ticks and 81.4% fleas (19.6% were co-infested with both ticks and fleas). In total, nine tick and three flea taxa were identified based on the ectoparasites’ DNA (accession numbers presented in Additional file 1: Table S2). For those habitat-country combinations with at least five infested individuals in each of the habitats: rural areas in Uganda (Shannon’s index [SI] = 1.57) and Namibia (SI = 1.55) had higher ectoparasite diversity compared with urban areas (SI Uganda = 1.23; SI Namibia = 0.60)—although their species frequency distributions did not differ in a statistically significant way (Fisher’s exact: *P* > 0.18). Species community distribution of Tanzania’s rural habitat (SI = 1.15) did not differ from urban habitat (SI = 1.22; Fisher’s exact: *P* = 0.98). Distributions of ectoparasite communities (fleas and tick species together, Additional file 1: Table S3) significantly differed among countries (Fisher’s exact tests; for all pairwise comparisons among countries *P* < 0.047), except for the comparison Namibia–Tanzania (*P* = 0.086) and Namibia–Uganda (*P* = 0.13).

### Tick infestation: prevalence and intensity

Within the subpopulation of ectoparasite-infested cats, substantial variation in tick prevalence was observed among countries (χ^2^ = 19.24, degrees of freedom [*df*] = 5, *P* = 0.0017), but we emphasize that—due to the dominance of fleas—tick prevalence outcomes should be interpreted with care and only for the subpopulation of ectoparasite-infested individuals. No statistically significant contrasts between habitat types were found in countries with at least five infested cats in each of the habitat types (Namibia, Tanzania, and Uganda).

**Fig. 2:**
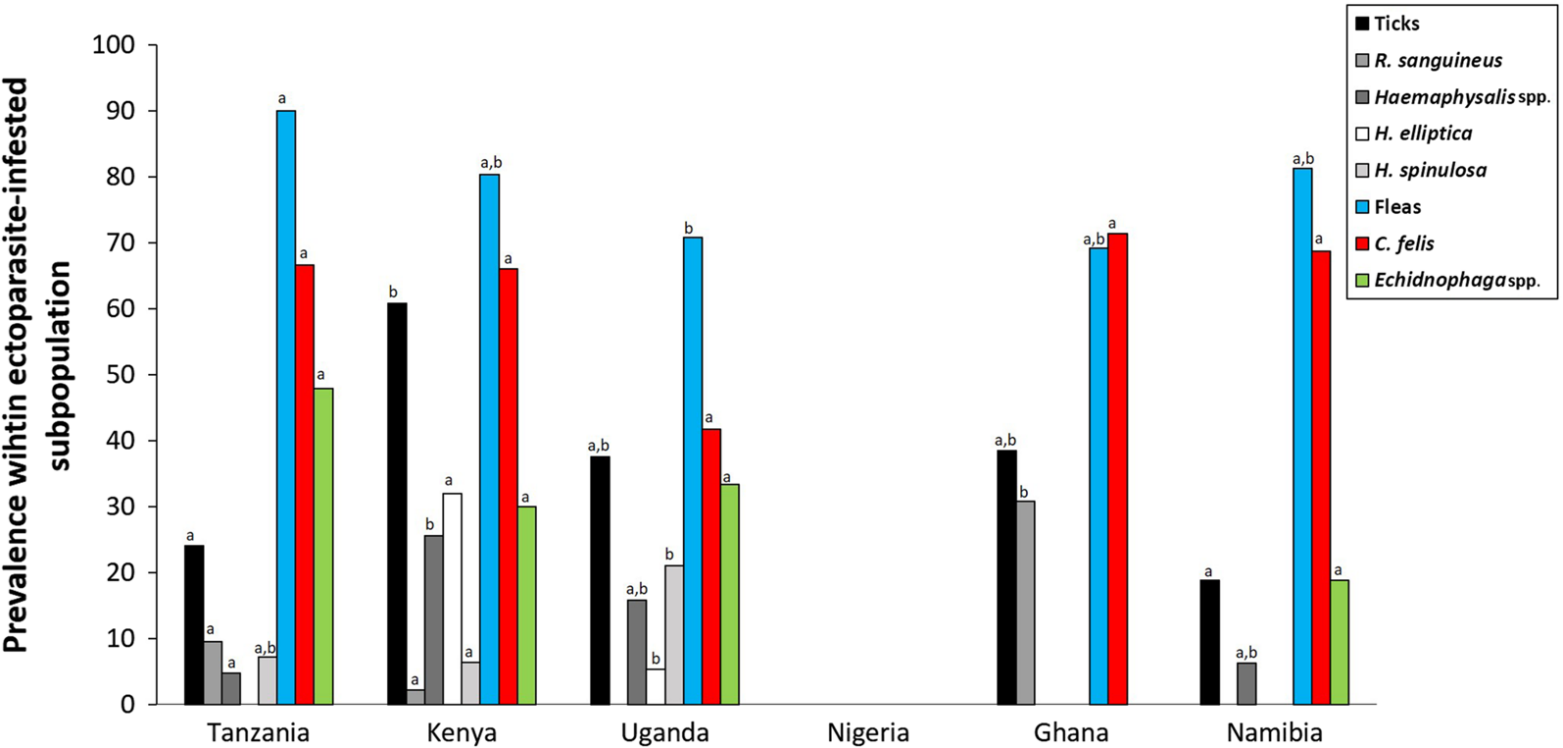
Macro-geographical variation in ectoparasite prevalence. Percentages within the population of infested cats, parasitized with the most common tick (grayscale) and flea (red, green, and blue) taxa (overall prevalence per taxon > 5%; see Table 3). For each taxon, if letters are the same (letters used: a, b, c, d) the contrast between countries is not statistically different from zero

**Fig. 3:**
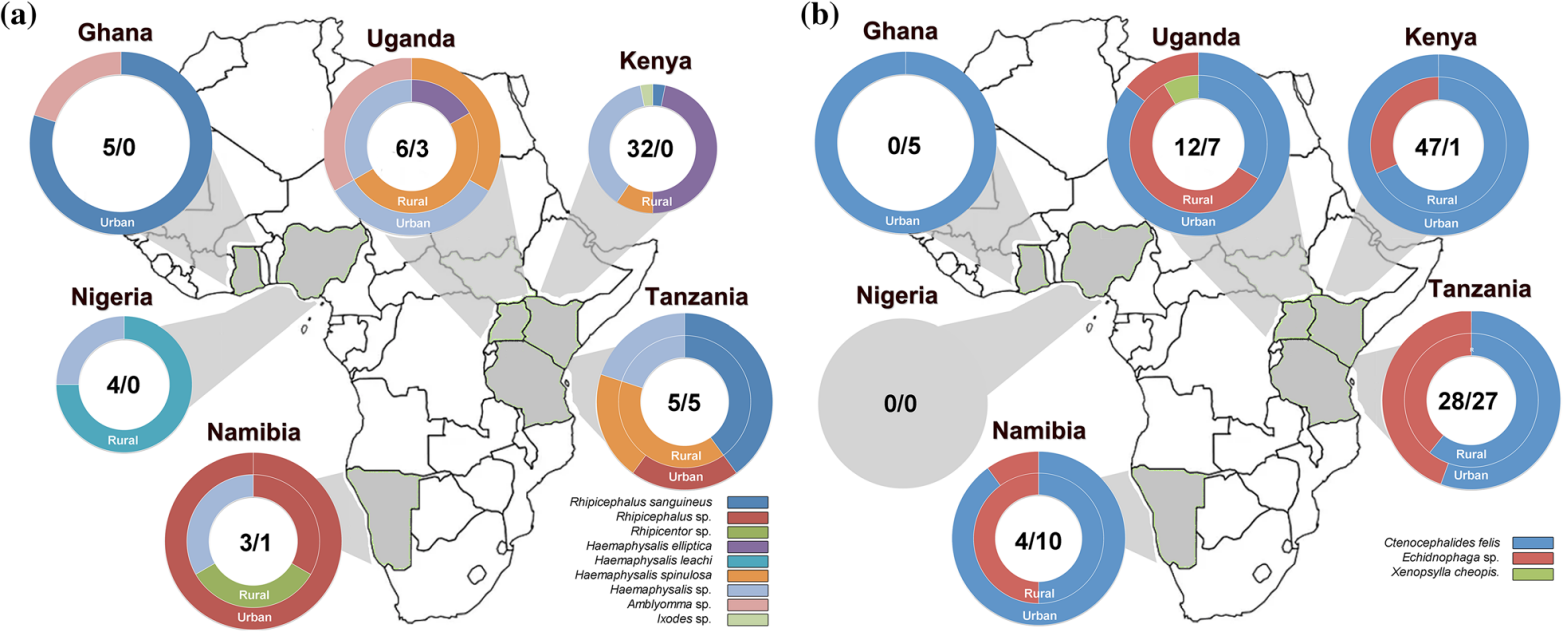
**a**, **b** Graphical overview of the tick (**a**) and flea (**b**) communities found in urban and rural areas of the six African countries joining the AFSCAN project (see Additional file 1: Table S3 for raw data). Numbers represent the PCR signals allocated to a tick taxon in the infested cats. Per cat, an extraction on a pooled set of ticks was done, before the PCR analysis was executed; a maximum of one PCR-positive per cat could be obtained for each of the taxa investigated

We refer to Table 3 and Figs. 2 and 3a for macro-geographical contrasts in prevalence at taxon level. *Haemaphysalis elliptica* (overall prevalence: 11.4%) was the most prevalent tick species but was only found in rural Kenya and Uganda. *Haemaphysalis spinulosa* (overall prevalence: 7.1%) was also found in Tanzania and urban Ghana. *Rhipicephalus sanguineus* sensu lato (s.l.) (overall prevalence: 6.4%) was isolated from cats in eastern (Ghana) and western Africa (Kenya and Tanzania). Unidentified *Haemaphysalis* spp*.* were collected from a larger proportion of the cats (13.5%), especially sampled in rural Kenya (25.5%). Contrasts in tick prevalence (rural vs. urban) for all the identified tick species did not differ significantly from zero in any of the countries. Among the individuals with co-infestations (10.3% of infested individuals), *H. elliptica* and *H. spinulosa* was the combination most frequently observed (5.2% overall), with the highest occurrence in rural Uganda (20.0%) (Additional file 1: Table S5).

**Table 3:** Tick and flea prevalence and intensity in infested cats of six African countries

	All	Tanzania (%)			Kenya (%)			Uganda (%)			Nigeria (%)			Ghana (%)			Namibia (%)		
		Rural	Urban	*P*	Rural	Urban	*P*	Rural	Urban	*P*	Rural	Urban	*P*	Rural	Urban	*P*	Rural	Urban	*P*
Ticks																			
*R. sanguineus* s.l.	6.4	8.7	10.5	ns	2.1			0.0	0.0		0.0				30.8		0.0	0.0	
*Rhipicephalus* sp.	2.1	0.0	5.3		0.0			0.0	0.0		0.0				0.0		20.0	9.1	ns
*Rhipicentor* sp.	0.7	0.0	0.0		0.0			0.0	0.0		0.0				0.0		20.0	0.0	ns
*H. elliptica*	11.4	0.0	0.0		31.9			7.1	0.0		0.0				0.0		0.0	0.0	
*H. leachi*	2.1	0.0	0.0		0.0			0.0	0.0		75.0				0.0		0.0	0.0	
*H. spinulosa*	7.1	8.7	5.3		6.4			21.4	20.0	ns	0.0				0.0		0.0	0.0	
*Haemaphysalis* sp.	13.5	4.4	5.3		25.5			14.3	20.0	ns	25.0				0.0		20.0	0.0	ns
*Amblyomma* sp.	1.4	0.0	0.0		0.0			0.0	20.0		0.0				7.7		0.0	0.0	
*Ixodes* sp.	0.7	0.0	0.0		2.1			0.0	0.0		0.0				0.0		0.0	0.0	
Tick	41.1	21.7	26.3	ns	61.7			35.7	40.0	ns	100.0				38.5		40.0	9.1	ns infestIND eq 'A'
Intensity		40.0	16.7		4.8			20.0	25.0		75.0				75.0		0.0	0.0	
Shannon’ index		1.1	1.3		1.2			1.0	1.1		0.6				0.5		1.1	0.0	
Fleas																			
*C. felis*	62.8	65.4	68.2	ns	65.3	100.00		28.6	60.0	ns	0.0				71.4		40.0	81.8	ns
*Echidnophaga* sp.	33.8	42.3	54.6	ns	30.6	0.00		50.0	10.0	*	0.0				0.0		40.0	9.1	ns
*Xenopsylla cheopis*	0.7	0.0	0.0		0.0	0.00		7.1	0.0		0.0				0.0		0.0	0.0	
Flea	81.4	84.6	95.5	ns	79.6	100.00		71.4	70.0	ns	0.0				71.4		60.0	90.9	ns
Intensity		5.3	0.0		3.3	0.00		0.0	0.0		0.0				0.0		0.0	0.0	
Shannon’ index		0.7	0.7		0.6	0.00		0.9	0.4		0.0				0.0		0.7	0.3	
Co-infestation	19.6	7.4	21.7		42.0	0.00		7.1	10.0		0.0				7.7		0.0	0.0	
Shannon’ index		1.2	1.2		1.52	0.00		1.6	1.2		0.6				0.9		1.6	0.6	
No. of cats*	158	27	23		50	1		14	10		4				13		5	11	

Ectoparasites were only identified molecularly. For each cat, a single extraction was made of a pooled set of ticks and/or fleas, and subsequently screened for the presence of DNA belonging to a particular tick and flea species. The identification was based on ITS1 or on 16S ribosomal mtDNA when the first was not yielding results. Next, the percentage of extracts (i.e., cats) containing DNA of a specific taxon was derived, within the population of infested cats. For statistical outcomes on pairwise macro-geographical differences, we refer to Fig. 2. Habitat differences (rural vs. urban) are investigated for countries with the presence of at least 10% in one of their habitats. As a measure of species diversity Shannon’s index and the accompanying significance level of Fisher’s exact test are provided. *P* < 0.05: *, *P* > 0.05: ns (not significant). No individuals were infested with *Rhipicephalus appendiculatus*, *R. simus, R. microplus*, or *R. senegalensis*, *A. variegatum* and *Echidnophaga gallinacea*

Overall, the among country variation in tick infestation intensity did significantly differ from zero (χ^2^ = 16.83, *df* = 5, *P* = 0.0048) with the proportion of the subpopulation having intermediate to high loads ranging from 0.0% (Namibia) to 75.0% (rural Nigeria and urban Ghana). Cats from Tanzania’s rural areas (40.0%) tended to have significantly higher tick infestation intensities than those from its urban areas (16.7%; χ^2^ = 3.49, *df* = 1, *P* = 0.06); no habitat differences were observed in those other countries that were considered to be sufficiently covered (Uganda and Namibia).

### Flea infestation: prevalence and intensity

Within the subpopulation of ectoparasite-infested cats, flea prevalence was much higher than tick prevalence for each of the countries (all *P*-values < 0.001). Among countries, variation in prevalence was high (range: 60.0–95.5%; χ^2^ = 18.89, *df* = 5, *P* = 0.0019). There were no significant habitat differences found in flea prevalence in any of the countries with at least five individuals sampled per habitat type.

Also, we found no statistically significant habitat contrasts in any flea identified at the taxon level, which might be due to underpowered tests (two-sided Fisher’s exact tests for all pairwise urban–rural comparisons *P* > 0.05), even not for *Echidnophaga* spp. in Uganda where rural areas (50.0%) tended to have more infested individuals than urban areas (10.0%; *P* = 0.08). *Xenopsylla cheopis* was observed only in rural Uganda (7.1%). *Ctenocephalides felis* and *Echidnophaga* sp. was logically the most frequently observed co-infestation (18.6% overall; see Additional file 1: Table S5). We refer to Table 3 and Figs. 2 and 3b for macro-geographical contrasts at the taxon level. *Ctenocephalides felis* (overall prevalence: 62.8%) was the most prevalent flea species, but was not found in Nigeria, where no flea infestations were observed on cats. The second most common flea species was *Echidnophaga* sp. (overall prevalence: 33.8%), also found in the same countries as *C. felis*. *Xenopsylla cheopsis*, with a very low overall prevalence of 0.7% was only found in Uganda as mentioned above.

The variation in flea infestation intensity (i.e., proportion of cats having intermediate to high loads, i.e., intensity > 10 fleas) was not very high (range 0.0–5.3%; χ^2^ = 5.20, *df* = 5, *P* = 0.27) and no statistically significant habitat contrasts were found in any of the countries.

### Vector-borne pathogens

The DNA of pathogens was detected in blood samples (three pathogen taxa), ticks (six taxa), and fleas (four taxa), and also antibodies against two pathogen genera. Below we report on the cat’s geographical occurrence in relation to the pathogen prevalence.

#### Pathogens in host blood

**Fig. 4:**
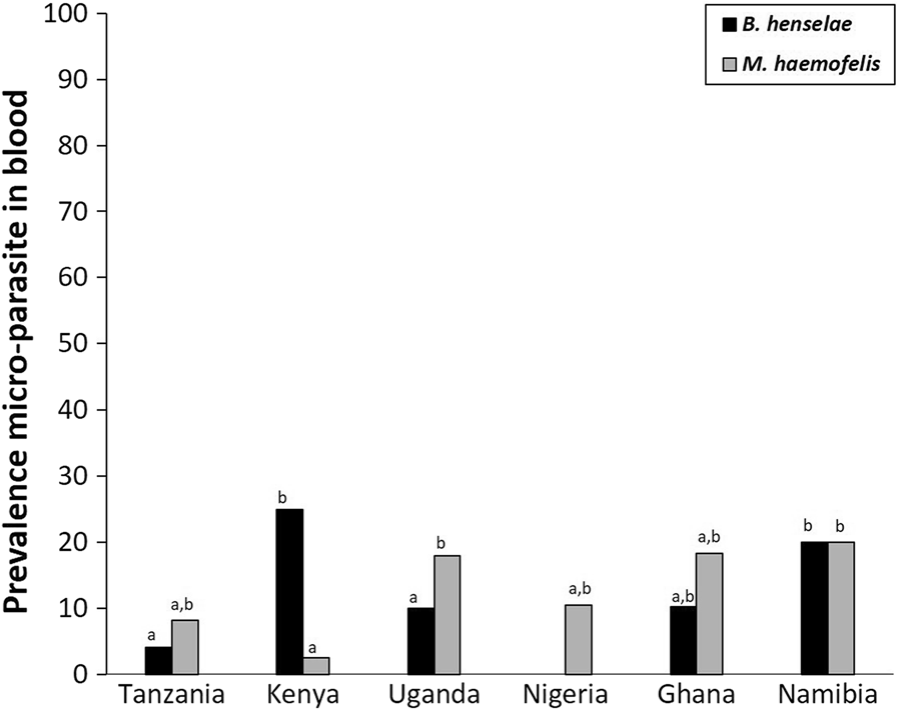
Macro-geographical variation in pathogen prevalence in cat blood, based on DNA screening. See legend Fig. 2 for interpretation of letters

In the blood samples of 276 individuals, *M. haemofelis* (overall 13.4%) was the most prevalent, and showed some variation among country–habitat combinations (range: 0.0–40.0%; χ^2^ = 10.71, *df* = 5, *P* = 0.06—note: only country–habitat combinations with at least five individuals). No consistent habitat differences in *M. haemofelis* prevalence could be deduced from the data; in Namibia rural areas showed the highest prevalence (χ^2^ = 5.35, *df* = 5, *P* = 0.02), while in Nigeria, this was the case for urban areas (χ^2^ = 5.43, *df* = 1, *P* = 0.02). Others did not show habitat differences at all (Uganda, Uganda, Tanzania; all *P*-values > 0.05). Also, for *Bartonella henselae* (overall prevalence: 11.6%) no habitat differences were found (see Table 4). It is worth mentioning that around a quarter of cats were infected with *B. henselae* in Kenya’s rural areas (25.6%) and Namibia’s urban areas (22.9%), while in all other country–habitat combinations prevalence were lower than 15.0%. No further analyses were performed on pathogens that were not detected or occurred in very low numbers. Seroconversion for the four pathogenic agents was rarely detected (*Borrelia* spp. 0.39%, and *Ehrlichia* spp. 0.39%). For an overview of prevalence data and contrasts between countries and/or habitats, we refer to Fig. 4 and Table 5, respectively.

**Table 4:** Pathogen prevalence in the blood of cats from six African countries

	Overall	Tanzania (%)		Kenya (%)		Uganda (%)		Nigeria (%)		Ghana (%)		Namibia (%)	
		Rural	Urban	Rural	Urban	Rural	Urban	Rural	Urban	Rural	Urban	Rural	Urban
Pathogens in blood													
*B. felis*	0.0	0.0	0.0	0.0	0.0	0.0	0.0	0.0	0.0	0.0	0.0	0.0	0.0
*B. henselae*	11.6	0.0	8.7ns	25.6	0.0	12.0	8.0ns	0.0	0.0	0.0	11.4ns	13.3	22.9ns
*M. haemofelis*	13.4	11.5	4.4ns	2.6	0.0	16.0	20.0ns	3.6	30.0*	0.0	20.5ns	40.0	11.4*
Individuals	276	26	23	39	1	25	25	28	10	5	44	15	35
Seropositive													
*Borrelia* spp.	0.4	0.0	0.0	0.0	0.0	0.0	4.0	0.0	0.0	0.0	0.0	0.0	0.0
*Ehrlichia* spp.	0.4	0.0	0.0	0.0	0.0	0.0	0.0	0.0	0.0	0.0	2.4	0.0	0.0
Individuals	257	22	23	28	1	25	25	28	10	5	42	14	34

DNA-based (qPCR) pathogen prevalence in the blood of cats from urban and rural areas of six African countries. In addition, seroprevalence of four pathogen genera is given, for which the IDEXX test was used. For statistical outcomes on pairwise macro-geographical differences, we refer to Fig. 4. As a measure of species diversity, Shannon’s index was provided. *P* < 0.05: *, *P* > 0.05: ns (not significant)

**Table 5:** Pathogen prevalence in flea and tick pools collected from infested cats

	Pathogens in ectoparasites	Overall	Tanzania (%)		Kenya (%)		Uganda (%)		Nigeria (%)		Ghana (%)		Namibia (%)	
			Rural	Urban	Rural	Urban	Rural	Urban	Rural	Urban	Rural	Urban	Rural	Urban
Ticks	*B. canis*	0.0	0.0	0.0	0.0		0.0	0.0	0.0			0.0	0.0	0.0
	*B. rossi*	1.7	0.0	0.0	3.5		0.0	0.0	0.0			0.0	0.0	0.0
	*E. chaffeensis*	0.0	0.0	0.0	0.0		0.0	0.0	0.0			0.0	0.0	0.0
	*A. platys*	1.7	0.0	0.0	0.0		0.0	25.0	0.0			0.0	0.0	0.0
	*A. phagocytophilum*	0.0	0.0	0.0	0.0		0.0	0.0	0.0			0.0	0.0	0.0
	*R. conorii*	1.7	0.0	0.0	0.0		20.0	0.0	0.0			0.0	0.0	0.0
	*R. africae*	5.0	0.0	0.0	3.5		0.0	25.0	0.0			20.0	0.0	0.0
	*C. burnetti*	8.3	20.0	0.0ns	6.9		0.0	0.0	25.0			20.0	0.0	0.0
	*H. canis*	50.0	80.0	40.0ns	55.2		0.0	25.0	75.0			40.0	100.0	0.0
	*D. immitis*	0.0	0.0	0.0	0.0		0.0	0.0	0.0			0.0	0.0	0.0
	*E. canis*	0.0	0.0	0.0	0.0		0.0	0.0	0.0			0.0	0.0	0.0
	*B. felis*	0.0	0.0	0.0	0.0		0.0	0.0	0.0			0.0	0.0	0.0
	Tick pools	60	5	5	29		5	4	4			5	2	1
	Shannon’ index		0.50	0.0	0.7	0.0	0.0	1.1	0.6			1.0	0.0	0.0
Fleas	*B. henselae*	11.9	4.6	14.3 ns	15.4	0.0	0.0	0.0				40.0	33.3	10.0
	*M. haemofelis*	10.2	0.0	14.3 ns	15.4	0.0	0.0	0.0				0.0	0.0	30.0
	*D. caninum*	5.1	0.0	0.0	7.7	0.0	0.0	0.0				20.0	33.3	10.0
	*R. felis*	100.0	100.0	100.0	100.0	100.0	100.0	100.0				100.0	100.0	100.0
	Flea pools	118	22	21	39	1	10	7				5	3	10
	Shannon’ index		0.0	0.7	1.1	0.00	0.00	0.00				0.6	0.7	1.0

**Fig. 5:**
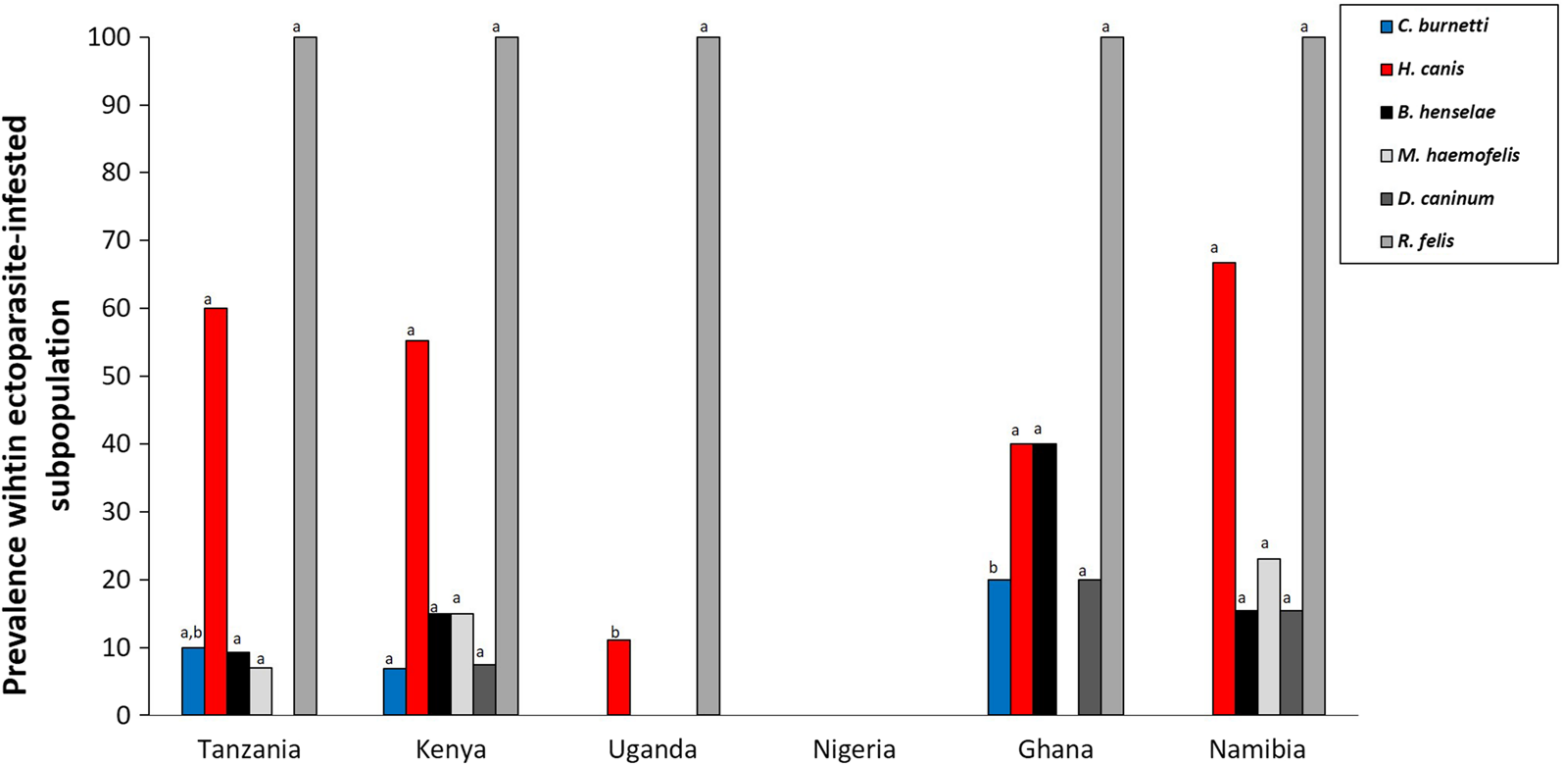
Macro-geographical variation in pathogen prevalence in ectoparasites isolated from cats. Percentages of pools of ticks collected from cats that are infected with one of the common flea-borne (grayscale) and tick-borne (red and blue) pathogens (overall prevalence > 5%; see Table 5). See legend Fig. 2 for interpretation of letters

Pathogen prevalence in 118 flea pools and 60 tick pools collected from infested cat individuals in urban and rural areas of six African countries. No pairwise comparisons were performed for countries in which fewer than five cat individuals were sampled in one of their habitats. For statistical outcomes on macro-geographical contrasts, we refer to Figs. 5. *P* > 0.05: ns (not significant)

#### Pathogens in ticks

In the ticks (60 pools screened), *Hepatozoon canis* (50.0%) was the most prevalent pathogen. *Coxiella burnetti* was found in 8.3% of the tick pools (Fig. 5). No habitat differences were found in Tanzania (where sample sizes allowed for a Fisher’s exact test) for either pathogen. Several other pathogens were detected in the ticks (*B. rossi*, *A. platys*, *R. conorii*, *R. africae*), though in (very) low numbers (Table 6). Overall, 13.9% of the infected ticks showed a co-infection (Additional file 1: Table S7).

**Table 6:** Vector-borne pathogen associations in ticks and fleas collected from cats

	*H. elliptica*	*Haemaphysalis* sp.	*C. felis*	*Echidnophaga* sp.
*H. canis*	53.9a	58.8a		
*R. africae*	0.0a	0.0a		
*C. burnetti*	0.0a	5.9a		
*B. henselae*			10.1a	11.1a
*D. caninum*			5.8a	3.7a
*M. haemofelis*			15.9a	0.0b
*R. felis*			100.0a	100.0a
*Samples*	13	17	69	27

A shared letter indicates no significant difference. Only the cats in which a single tick taxon was observed (based on the extractions of the set of pooled ticks) were included

#### Pathogens in fleas

*Rickettsia felis* was detected in all 118 flea pools, despite the pathogen not being found in the cats’ blood. *Bartonella henselae* (overall prevalence: 11.9%) and *M. haemofelis* (overall prevalence: 10.2%) infections were found in all countries, except Uganda and Ghana (despite its presence in the cats’ blood) (Fig. 5). *Dipylidium caninum* DNA was detected in fleas from Kenya, Namibia and Ghana. No habitat differences were observed in any of the infection patterns (see Table 5). We refer to Additional file 1: Table S8 for co-infections.

#### Pathogen-tick associations

An explicit analysis of those pools of ticks in which the DNA of only a single species was detected (Table 6) showed that the occurrence of *H. canis* was not solely linked to *H. elliptica* (55.9%); also, the unidentified *Haemaphysalis* sp. showed an equally high prevalence (58.8%). *Bartonella henselae*, *D. caninum*, and *R. felis* were each equally linked to the flea taxa *C. felis* and *Echidnophaga* sp. In contrast, *M. haemofelis* showed a significantly higher affinity for *C. felis* (15.9% of the 69 flea pools) than *Echidnophaga* sp. (0% of the 27 pools; χ^2^ = 4.86, *df* = 1, *P* = 0.03).

## Discussion

The main objective of the study was to identify the most prevalent ectoparasites and vector-borne pathogens of cats in six sub-Saharan African countries. We conducted the data and sample collection via a rigorous predefined protocol and conducted a meta-analysis on standardized data. The presence of ticks, fleas, and pathogens in both vector and host was examined, concentrating on strong differences between broad country–specific urban categories (see “Methods” section). Numerous studies on ticks and their pathogens have been described in Africa, but these mainly focus on production animals in farming areas [7]. This study shows that, despite companion animals being of significant socioeconomic value to humans, they entail a risk for the spread of feline pathogens and increased risk of zoonotic vector-borne diseases in a One Health perspective by hosting pathogenic agents and their vectors. Where cats are in close contact with wildlife and production animals as cattle, they are at the interface of several vertebrate communities, however with different functionalities to humans.

Overall, 41% of the infested cats carried ticks, which is significantly lower than that of the sympatric dogs that were sampled in the same period [2]. In comparison to dogs, the overall prevalence in the subpopulation of infested animals was 95%. Most likely, the grooming behavior of cats favors tick removal but less efficiently flea removal. Nevertheless, similar tick species were found on cats compared with dogs. *Rhipicephalus sanguineus* s.l., commonly known as the brown dog tick [8, 9], is found worldwide in warmer climates and is a monotropic (dogs) three-host tick and mainly associated with man-made structures [2]. Apart from feeding on dogs, this species was commonly found on cats, being carnivores as well, but mainly in Tanzania and Ghana. In the latter country, this tick was only found in urban areas whereas in Tanzania it occurred in both habitat types. The lower prevalence of *R. sanguineus* s.l. on cats compared with dogs could be explained by the behavior of the cat itself. As dogs show a more endophilic behavior, being closely related to man made structures, just like the dog tick, dogs are more prone to come in contact with questing *R. sanguineus* s.l. stages. Cats, however, are more exophilic and might not be exposed to this species to the same extent. Considering the host specificity of *R. sanguineus* s.l., dogs are the preferred host although other carnivores like cats can be infested as well [9]. In Namibia and Tanzania, unidentified ticks belonging to this genus were identified with a presence in both rural and urban areas. These ticks could not be identified, as no similar sequence data were found in GenBank on ITS1 or 16S. Further morphological and molecular research is needed to clarify the status of these ticks.

Ticks of the *Haemaphysalis* genus where significantly more abundant compared with *Rhipicephalus* species. Many ticks of the *Haemaphysalis* genus, are linked to wildlife and considered exophilic [10]. As a consequence, several identified (*H. leachi*, *H. elliptica*, and *H. spinulosa*) and undefined *Haemaphysalis* ticks were observed more often on cats from rural than urban areas. In *H. leachi* and *H. elliptica* (previously classified as *H. leachi* as well [11]), adults parasitize domestic and wild carnivores, whilst the immature stages feed on rodents. *Haemaphysalis spinulosa* adults appear to feed on various small and medium-sized carnivores, as well as hedgehogs [11]. A very high proportion of individuals belonging to the genus *Haemaphysalis* could not be identified, but genetic clustering revealed a large group taxon, that most likely involves a new or genetically uncharacterized species (manuscript in preparation).

Flea infestations occurred on almost half of the cats sampled, and twice as many cats were infested with fleas compared with ticks. The predominant species *C. felis*, the cat flea, accounted for 65% of the infestations followed by the sticktight fleas *Echidnophaga* sp., both prevalent in all countries sampled. In comparison to fleas on dogs in the same area [2], dogs were hardly infested with *Echidnophaga* sp. As this flea species was commonly found in rural areas, with a host preference for fowl, cats might become more exposed due to their exophilic behavior. The oriental rat flea *Xenopsylla cheopis* was only found on cats in Uganda and Namibia. As this flea is the primary vector for bubonic plague and murine typhus, it poses a zoonotic risk to owners of cats.

Ten vector-borne pathogens were detected in host blood and/or vectors. Looking at the flea and pathogen correlation, only for *M. haemofelis* a vector-specific link was observed with the cat flea *C. felis*, whereas *B. henselae*, *D. caninum*, and *R. felis* were identified in both *C. felis* and *Echidnophaga* sp. but without indication of any causative correlation or vector competence. All flea pools were however infected with *R. felis* meaning that any cat with a flea infestation carries pathogens with zoonotic potential.

When considering the distributions of the pathogens with respect to tick species, the most striking contrast is the high prevalence of *C. burnetti* in the genus *Haemaphysalis* in which all members of the genus are known to be vector-competent for this pathogen [12]. No blood samples of cats were screened for this pathogen so no correlation could be determined. The occurrence of *H. canis* did not show a strong preference for a particular tick species, indicating that both genera could equally contribute to *H. canis*’ transmission—via ingestion of infected ticks.

*Borrelia* seroprevalence was extremely low and only one positive cat was found in Uganda, likely because of the low prevalence of *Ixodes* species, the main vectors of *Borrelia burgdorferi* s.l. [13]. Few cats were also found seropositive for *Ehrlichia* sp. (*E. canis* or *E. ewingii*) in Ghana, and its vector *R. sanguineus* s.l. was most abundant in the country compared with the other countries. It must be noted that the IDEXX 4D Plus is specifically developed for dogs and is less sensitive for screening cats [14].

In addition to the study on dogs in the same area [2], this work forms the most extensive and standardized study in sub-Saharan countries so far on cats, giving an overview of important vectors and vector-borne pathogens and which could serve as baseline data for future research and interventions.

In the previous and the current study, we found tick species and pathogens that are not classically associated with companion animals but still with the potential to transmit zoonotic disease-causing pathogens in dogs and cats. Significant levels of co-infestation and co-infections were observed, adding to the zoonotic risk, given the high potential of bridging opportunities to production animals and humans via vectors and/or immunomodulation and atypical virulence patterns (due to co-parasitism). Furthermore, we found multi-host ticks in urban areas, which have the potential to extend the network of pathogen transmission to humans.

## Conclusions

This large-scale and standardized study highlights the importance of ectoparasites and the pathogens of cats in sub-Saharan Africa, with co-parasitism being the rule rather than the exception. This information regarding companion animals, in combination with the more available information on pathogens of production animals and wildlife, would allow a holistic view of the risk the different host species, including humans, are exposed to. Furthermore, species-specific responses to space characteristics [15] in least-cost path-setting making use of habitat connectivity, will substantially increase our understanding of how spatial elements could affect local vector-borne pathogen risk. Integration of this knowledge with a good understanding of current complexities in socioeconomic and climate changes will enable policymakers and scientists to provide prevention strategies.

## Supplementary Information

**Additional file 1: Figure S1.** Overview of the moments of sampling within the average seasonal variation in precipitation and temperature. **Table S1.** Different multiplex assays for the detection of tick- and flea-borne pathogens. **Table S2**. Accession numbers for tick and flea species identified. **Table S3.** Distribution of PCR signals allocated to an ectoparasite taxon (identification at genus level and more precise) in the infested cats of urban and rural areas. **Table S4.** Distribution of co-infested cats within the subpopulation of tick-infested cats. **Table S5.** Co-infestations by different flea species (identification at genus level and lower). **Table S6.** Co-infections in cat blood. **Table S7.** Co-infections in cat ticks. **Table S8.** Co-infections in cat fleas.

**Additional file 2.** Capture form.

## Data Availability

The datasets used and/or analyzed during the current study are available from the corresponding author on reasonable request. The newly generated sequences were submitted to GenBank under accession numbers OP143941–OP143962 for the tick and flea species identified (*Ctenocephalis felis*, *Rhipicephalus sanguineus*, *Haemaphysalis leachi*, *H. spinulosa*, *Rhipicentor nuttalli*).

## Abbreviations

BLAST: Basic local alignment search tool
DNA: Deoxyribonucleic acid
GEE: Generalized estimation equation model
mtDNA: Mitochondrial DNA
qPCR: Quantitative real-time polymerase chain reaction
