# Supplementary material for: A community approach for pathogens and their arthropod vectors (ticks and fleas) in cats of sub-Saharan Africa
Source: Parasit Vectors. 2022 Sep 9;15:321. doi: 10.1186/s13071-022-05436-y (PMC9461260; doi:10.1186/s13071-022-05436-y)
Supplement: Supplementary file 1 — Additional file 1: Figure S1. Overview of the moments of sampling within the average seasonal variation in precipitation and temperature. Table S1. Different multiplex assays for the detection of tick- and flea-borne pathogens. Table S2. Accession numbers for tick and flea species identified. Table S3. Distribution of PCR signals allocated to an ectoparasite taxon (identification at genus level and more precise) in the infested cats of urban and rural areas. Table S4. Distribution of co-infested cats within the subpopulation of tick-infested cats. Table S5. Co-infestations by different flea species (identification at genus level and lower). Table S6. Co-infections in cat blood. Table S7. Co-infections in cat ticks. Table S8. Co-infections in cat fleas. [file 13071_2022_5436_MOESM1_ESM.docx]

**
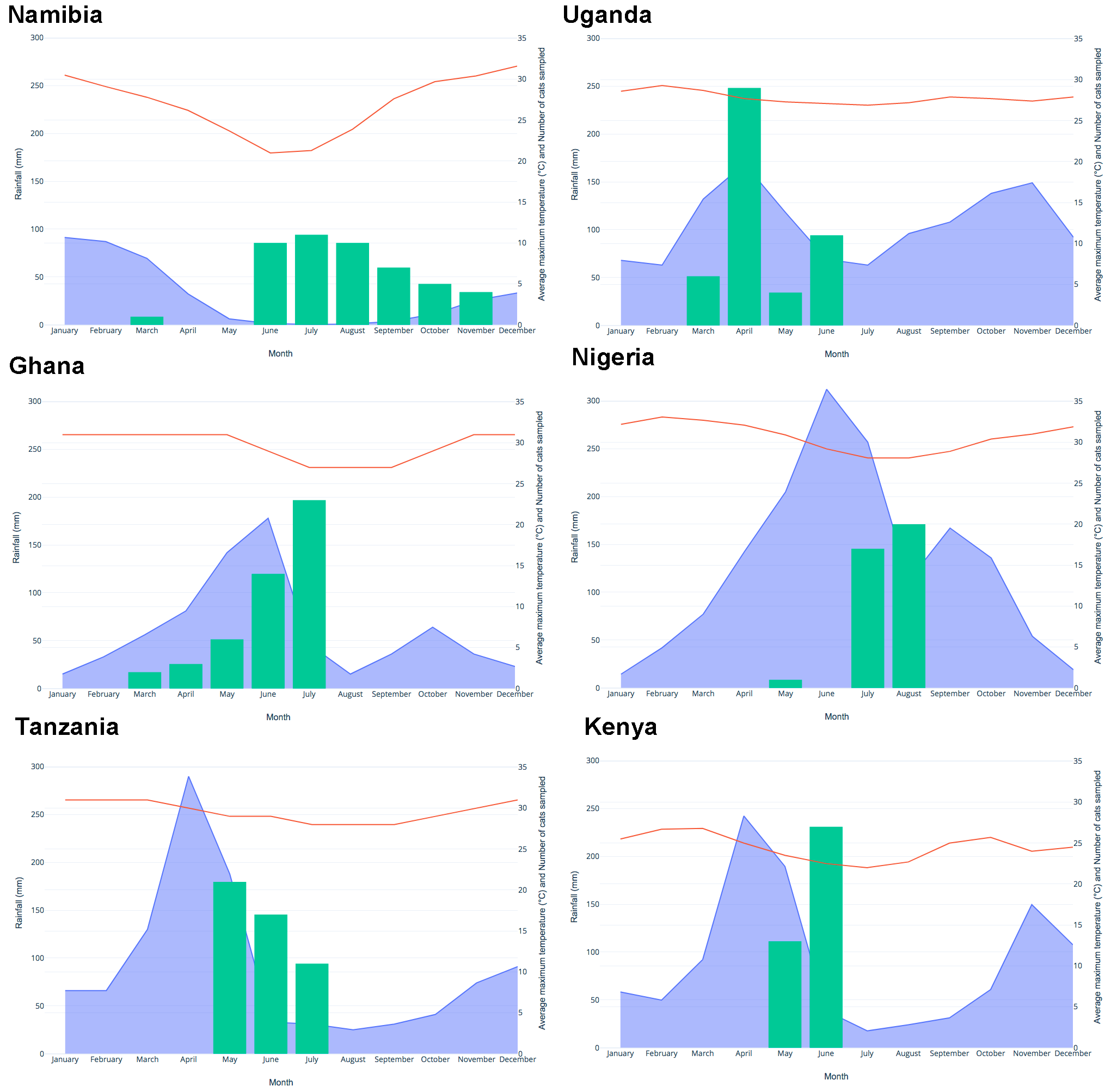
**

**Fig. S1:** Overview of the moments of sampling within the average seasonal variation in precipitation and temperature (data obtained from https://weather-and-climate.com). Except for Namibia, the majority of samples were retrieved during the rainy season.

**Table S1.** Different multiplex assays for the detection of tick- and flea-borne pathogens.

| **Multiplex 1** | Sample type |
| --- | --- |
| *Babesia rossi* | Ticks |
| *Babesia canis* |  |
| *Ehrlichia chaffeensis* |  |
| **Multiplex 2** |  |
| *Anaplasma phagocytophilum* | Ticks |
| *Anaplasma platys* |  |
| *Rickettsia africae* |  |
| *Rickettsia conorri* |  |
| **Multiplex 3** |  |
| *Coxiella burnetti* | Ticks |
| *Dirofilaria immitis* |  |
| **Multiplex 4** |  |
| *Babesia felis* | Feline blood and fleas |
| *Rickettsia felis* |  |
|  |  |
| *Ehrlichia canis* | Ticks |
|  |  |
| *Bartonella henselae* | Feline blood and fleas |
|  |  |
| *Mycoplasma haemofelis* | Feline blood and fleas |
|  |  |
| *Dipylidium caninum* | Fleas |

**Table S2.** Accession numbers for tick and flea species identified.

| Tick/flea ID | GenBank accession number | Newly generated accession numbers | Sequence identity of the obtained sequence to closest-matched accession number |
| --- | --- | --- | --- |
| *R. sanguineus* | JQ737127.1 | OP143948-OP143952 | 89.8-91.5% |
| *C. felis* | HF583247 | OP143941-OP143947 | 90.3-92.4% |
| *Rhipicephalus* sp. | JQ737127.1 |  | 82.2-88.8% |
| *Rhipicentor* sp. | MF818019.1 | OP143961-OP143962 | 82.3% |
| *Haemaphysalis* sp. | In-house sequence data |  | 80-90% |
| *Echidnophaga* sp. | EU169199 |  | 89-90.1% |
| *Amblyomma* sp. | KY457490 |  | 85.3-87.5% |
| *Ixodes* spp. | GU318946; GU318946 |  | 82.5% |
| *Haemaphysalis elliptica* | In-house sequence data |  | 90-90.3% |
| *Xenopsylla cheopis* | DQ295058 |  | 91.9% |
| *Haemaphysalis spinulosa* | KJ613637 | OP143956-OP143960 | 89.9-92.46% |
| *Haemaphysalis leachi* | MN661151 | OP143953-OP143955 | 92.2-92.4% |

**Table S3.** Distribution of PCR-signals allocated to an ectoparasite taxon (identification at genus level and more precise) in the infested cats of urban and rural areas in six African countries (Percentages sum to 100% for each country x habitat combination).

|  |  |  | Tanzania (%) | | Kenya (%) | | Uganda (%) | | Nigeria (%) | | Ghana (%) | | Namibia (%) | |
| --- | --- | --- | --- | --- | --- | --- | --- | --- | --- | --- | --- | --- | --- | --- |
|  | Species | Overall | Rural | Urban | Rural | Urban | Rural | Urban | Rural | Urban | Rural | Urban | Rural | Urban |
| Ticks | *R_sanguineus* | 14.1 | 40.0 | 40.0 | 3.1 | 0.0 | 0.0 | 0.0 | 0.0 | 0.0 | 0.0 | 80.0 | 0.0 | 0.0 |
|  | *Rhipicephalus* sp*.* | 4.7 | 0.0 | 20.0 | 0.0 | 0.0 | 0.0 | 0.0 | 0.0 | 0.0 | 0.0 | 0.0 | 33.3 | 100.0 |
|  | *Rhipicentor* sp. | 1.6 | 0.0 | 0.0 | 0.0 | 0.0 | 0.0 | 0.0 | 0.0 | 0.0 | 0.0 | 0.0 | 33.3 | 0.0 |
|  | *H_elliptica* | 25.0 | 0.0 | 0.0 | 46.9 | 0.0 | 16.7 | 0.0 | 0.0 | 0.0 | 0.0 | 0.0 | 0.0 | 0.0 |
|  | *H_leachi* | 4.7 | 0.0 | 0.0 | 0.0 | 0.0 | 0.0 | 0.0 | 75.0 | 0.0 | 0.0 | 0.0 | 0.0 | 0.0 |
|  | *H_spinulosa* | 15.6 | 40.0 | 20.0 | 9.4 | 0.0 | 50.0 | 33.3 | 0.0 | 0.0 | 0.0 | 0.0 | 0.0 | 0.0 |
|  | *Haemaphysalis* sp. | 29.7 | 20.0 | 20.0 | 37.5 | 0.0 | 33.3 | 33.3 | 25.0 | 0.0 | 0.0 | 0.0 | 33.3 | 0.0 |
|  | *Amblyomma* sp*.* | 3.1 | 0.0 | 0.0 | 0.0 | 0.0 | 0.0 | 33.3 | 0.0 | 0.0 | 0.0 | 20.0 | 0.0 | 0.0 |
|  | *Ixodes* sp*.* | 1.6 | 0.0 | 0.0 | 3.1 | 0.0 | 0.0 | 0.0 | 0.0 | 0.0 | 0.0 | 0.0 | 0.0 | 0.0 |
|  | Tick-infested cats | 64 | 5 | 5 | 32 | 0 | 6 | 3 | 4 | 0 | 0 | 5 | 3 | 1 |
|  |  |  |  |  |  |  |  |  |  |  |  |  |  |  |
| Fleas | *C. felis* | 64.5 | 60.7 | 55.6 | 68.1 | 100.0 | 33.3 | 85.7 | 0.0 | 0.0 | 0.0 | 100.0 | 50.0 | 90.0 |
|  | *Echidnophaga* sp. | 34.8 | 39.3 | 44.4 | 31.9 | 0.0 | 58.3 | 14.3 | 0.0 | 0.0 | 0.0 | 0.0 | 50.0 | 10.0 |
|  | *X. cheopis* | 0.7 | 0.0 | 0.0 | 0.0 | 0.0 | 8.3 | 0.0 | 0.0 | 0.0 | 0.0 | 0.0 | 0.0 | 0.0 |
|  | Flea-infested cats | 141 | 28 | 27 | 47 | 1 | 12 | 7 | 0 | 0 | 0 | 5 | 4 | 10 |
|  |  |  |  |  |  |  |  |  |  |  |  |  |  |  |
|  | | | | | | | | | | | | | | |

Note: Per cat, an extraction on a pooled set of ticks and fleas was done, before the PCR-analysis was executed; maximum one PCR-positive per cat could be obtained for each of the taxa investigated.

In bold prevalence for tick batches ≥ 5 individuals

**Table S4.** Distribution of co-infested cats within the subpopulation of tick-infested cats.

|  |  | Tanzania (%) | | Kenya (%) | | Uganda (%) | | Nigeria (%) | | Ghana (%) | | Namibia (%) | |
| --- | --- | --- | --- | --- | --- | --- | --- | --- | --- | --- | --- | --- | --- |
| Species and co-infestations | Overall | Rural | Urban | Rural | Urban | Rural | Urban | Rural | Urban | Rural | Urban | Rural | Urban |
| *Haemaphysalis* sp. | 29.3 | 20.0 | 20.0 | 37.9 | 0.0 | 40.0 | 50.0 | 25.0 | 0.0 | 0.0 | 0.0 | 0.0 | 0.0 |
| *H. elliptica* | 22.4 | 0.0 | 0.0 | 44.8 | 0.0 | 0.0 | 0.0 | 0.0 | 0.0 | 0.0 | 0.0 | 0.0 | 0.0 |
| *R. sanguineus* | 13.8 | 40.0 | 40.0 | 0.0 | 0.0 | 0.0 | 0.0 | 0.0 | 0.0 | 0.0 | 80.0 | 0.0 | 0.0 |
| *H_spinulosa* | 10.3 | 40.0 | 20.0 | 3.5 | 0.0 | 40.0 | 0.0 | 0.0 | 0.0 | 0.0 | 0.0 | 0.0 | 0.0 |
| *H. elliptica* and *H. spinulosa* | 5.2 | 0.0 | 0.0 | 6.9 | 0.0 | 20.0 | 0.0 | 0.0 | 0.0 | 0.0 | 0.0 | 0.0 | 0.0 |
| *H_leachi* | 5.2 | 0.0 | 0.0 | 0.0 | 0.0 | 0.0 | 0.0 | 75.0 | 0.0 | 0.0 | 0.0 | 0.0 | 0.0 |
| *Rhipicephalus* sp. | 5.2 | 0.0 | 20.0 | 0.0 | 0.0 | 0.0 | 0.0 | 0.0 | 0.0 | 0.0 | 0.0 | 50.0 | 100.0 |
| *Amblyomma* sp. | 1.7 | 0.0 | 0.0 | 0.0 | 0.0 | 0.0 | 0.0 | 0.0 | 0.0 | 0.0 | 20.0 | 0.0 | 0.0 |
| *Amblyomma* sp. and *H. spinulosa* | 1.7 | 0.0 | 0.0 | 0.0 | 0.0 | 0.0 | 50.0 | 0.0 | 0.0 | 0.0 | 0.0 | 0.0 | 0.0 |
| *Haemaphysalis* sp. and *R. sanguineus* | 1.7 | 0.0 | 0.0 | 3.5 | 0.0 | 0.0 | 0.0 | 0.0 | 0.0 | 0.0 | 0.0 | 0.0 | 0.0 |
| *Haemaphysalis* sp. and *Rhipicentor* sp. | 1.7 | 0.0 | 0.0 | 0.0 | 0.0 | 0.0 | 0.0 | 0.0 | 0.0 | 0.0 | 0.0 | 50.0 | 0.0 |
| *Ixodes* sp. | 1.7 | 0.0 | 0.0 | 3.5 | 0.0 | 0.0 | 0.0 | 0.0 | 0.0 | 0.0 | 0.0 | 0.0 | 0.0 |
| Co-infested cats (%) | 10.3 | 0.0 | 0.0 | 10.4 | 0.0 | 20.0 | 50.0 | 0.0 | 0.0 | 0.0 | 0.0 | 50.0 | 0.0 |
| Infested cats | 58 | 5 | 5 | 29 | 0 | 5 | 2 | 4 | 0 | 0 | 5 | 2 | 1 |
|  |  |  |  |  |  |  |  |  |  |  |  |  |  |

Note: Percentages refer to cats with one or more tick species feeding on them. From top to bottom: highest to lowest combinations observed. Identification at genus level and lower (Percentages sum to 100% for each country x habitat combination).

**Table S5.** Infestations and co-infestations by different flea species (identification at genus level and lower).

|  |  | Tanzania (%) | | Kenya (%) | | Uganda (%) | | Nigeria (%) | | Ghana (%) | | Namibia (%) | |
| --- | --- | --- | --- | --- | --- | --- | --- | --- | --- | --- | --- | --- | --- |
| Infestations/co-infestations | Overall | Rural | Urban | Rural | Urban | Rural | Urban | Rural | Urban | Rural | Urban | Rural | Urban |
| *C. felis* | 58.5 | 50.0 | 42.9 | 61.5 | 100.0 | 30.0 | 85.7 | 0.0 | 0.0 | 0.0 | 100.0 | 33.3 | 90.0 |
| *Echidnophaga* sp. | 22.0 | 22.7 | 28.6 | 18.0 | 0.0 | 50.0 | 14.3 | 0.0 | 0.0 | 0.0 | 0.0 | 33.3 | 10.0 |
| *C. felis* and *Echidnophaga* sp. | 18.6 | 27.3 | 28.6 | 20.5 | 0.0 | 10.0 | 0.0 | 0.0 | 0.0 | 0.0 | 0.0 | 33.3 | 0.0 |
| *X. cheopis* and *Echidnophaga* sp. | 0.9 | 0.0 | 0.0 | 0.0 | 0.0 | 10.0 | 0.0 | 0.0 | 0.0 | 0.0 | 0.0 | 0.0 | 0.0 |
| Co-infested individuals (%) | 19.5 | 27.3 | 28.6 | 20.5 | 0.0 | 20.0 | 0.0 | 0.0 | 0.0 | 0.0 | 0.0 | 33.3 | 0.0 |
| Infested cats | 118 | 22 | 21 | 39 | 1 | 10 | 7 | 0 | 0 | 0 | 5 | 3 | 10 |
|  | | | | | | | | | | | | | |

Note: Prevalences refer to the number of host individuals with one or more flea species feeding on them. From top to bottom: highest to lowest combinations observed.

**Table S6.** Infections and co-infections in cat blood.

|  |  | Tanzania (%) | | Kenya (%) | | Uganda (%) | | Nigeria (%) | | Ghana (%) | | Namibia (%) | |
| --- | --- | --- | --- | --- | --- | --- | --- | --- | --- | --- | --- | --- | --- |
| Infections/co-infections | Overall | Rural | Urban | Rural | Urban | Rural | Urban | Rural | Urban | Rural | Urban | Rural | Urban |
| *E. canis* | 2.9 | 0.0 | 0.0 | 0.0 | 0.0 | 0.0 | 0.0 | 0.0 | 0.0 | 0.0 | 6.3 | 0.0 | 7.7 |
| *H. canis* | 2.9 | 0.0 | 0.0 | 0.0 | 0.0 | 0.0 | 0.0 | 0.0 | 0.0 | 0.0 | 12.5 | 0.0 | 0.0 |
| *H. canis* and *D. immitis* | 1.5 | 0.0 | 0.0 | 0.0 | 0.0 | 0.0 | 0.0 | 0.0 | 0.0 | 0.0 | 6.3 | 0.0 | 0.0 |
| *H. canis* and *E. canis* | 1.5 | 0.0 | 0.0 | 0.0 | 0.0 | 0.0 | 0.0 | 0.0 | 0.0 | 0.0 | 6.3 | 0.0 | 0.0 |
|  |  |  |  |  |  |  |  |  |  |  |  |  |  |
| Co-infected individuals (%) | 2.9 | 0.0 | 0.0 | 0.0 | 0.0 | 0.0 | 0.0 | 0.0 | 0.0 | 0.0 | 12.5 | 0.0 | 0.0 |
| Blood samples with at least one pathogen | 68 | 3 | 3 | 10 | 0 | 6 | 6 | 1 | 3 | 0 | 16 | 7 | 13 |
|  | | | | | | | | | | | | | |

Note: Prevalences refer to the number of host individuals with one or more pathogens. From top to bottom: highest to lowest prevalence observed.

**Table S7.** Infections and co-infections in cat ticks.

|  |  | Tanzania (%) | | Kenya (%) | | Uganda (%) | | Nigeria (%) | | Ghana (%) | | Namibia (%) | |
| --- | --- | --- | --- | --- | --- | --- | --- | --- | --- | --- | --- | --- | --- |
| Infections/co-infections | Overall | Rural | Urban | Rural | Urban | Rural | Urban | Rural | Urban | Rural | Urban | Rural | Urban |
| *H. canis* | 72.2 | 75.0 | 100.0 | 84.2 | 0.0 | 0.0 | 0.0 | 66.7 | 0.0 | 0.0 | 33.3 | 100.0 | 0.0 |
| *C. burnetti* | 5.6 | 0.0 | 0.0 | 5.3 | 0.0 | 0.0 | 0.0 | 0.0 | 0.0 | 0.0 | 33.3 | 0.0 | 0.0 |
| *C. burnetti* and *H. canis* | 5.6 | 25.0 | 0.0 | 0.0 | 0.0 | 0.0 | 0.0 | 33.3 | 0.0 | 0.0 | 0.0 | 0.0 | 0.0 |
| *R. africae* and *H. canis* | 5.6 | 0.0 | 0.0 | 0.0 | 0.0 | 0.0 | 50.0 | 0.0 | 0.0 | 0.0 | 33.3 | 0.0 | 0.0 |
| *A. platys* | 2.8 | 0.0 | 0.0 | 0.0 | 0.0 | 0.0 | 50.0 | 0.0 | 0.0 | 0.0 | 0.0 | 0.0 | 0.0 |
| *B. rossi* | 2.8 | 0.0 | 0.0 | 5.3 | 0.0 | 0.0 | 0.0 | 0.0 | 0.0 | 0.0 | 0.0 | 0.0 | 0.0 |
| *R. africae* and *C. burnetti* | 2.8 | 0.0 | 0.0 | 5.3 | 0.0 | 0.0 | 0.0 | 0.0 | 0.0 | 0.0 | 0.0 | 0.0 | 0.0 |
| *R. conorii* | 2.8 | 0.0 | 0.0 | 0.0 | 0.0 | 100.0 | 0.0 | 0.0 | 0.0 | 0.0 | 0.0 | 0.0 | 0.0 |
|  |  |  |  |  |  |  |  |  |  |  |  |  |  |
| Co-infected individuals (%) | 13.9 | 25.0 | 0.0 | 5.3 | 0.0 | 0.0 | 50.0 | 33.3 | 0.0 | 0.0 | 33.3 | 0.0 | 0.0 |
| N° of infested cats with at least one pathogen found in the tick pool | 36 | 4 | 2 | 19 | 0 | 1 | 2 | 3 | 0 | 0 | 3 | 2 | 0 |
|  | | | | | | | | | | | | | |

Note: Prevalences refer to tick pools (given extractions at cat individual level have been obtained of a pool of ticks) with one or more pathogen species. From top to bottom: highest to lowest prevalences observed.

**Table S8.** Infections and co-infections in cat fleas.

|  |  | Tanzania (%) | | Kenya (%) | | Uganda (%) | | Nigeria (%) | | Ghana (%) | | Namibia (%) | |
| --- | --- | --- | --- | --- | --- | --- | --- | --- | --- | --- | --- | --- | --- |
| Infections/co-infections | Overall | Rural | Urban | Rural | Urban | Rural | Urban | Rural | Urban | Rural | Urban | Rural | Urban |
| *B. henselae* | 35.7 | 100.0 | 25.0 | 30.8 | 0.0 | 0.0 | 0.0 | 0.0 | 0.0 | 0.0 | 66.7 | 50.0 | 20.0 |
| *M. haemofelis* | 35.7 | 0.0 | 25.0 | 46.2 | 0.0 | 0.0 | 0.0 | 0.0 | 0.0 | 0.0 | 0.0 | 0.0 | 60.0 |
| *D. caninum* | 14.3 | 0.0 | 0.0 | 7.7 | 0.0 | 0.0 | 0.0 | 0.0 | 0.0 | 0.0 | 33.3 | 50.0 | 20.0 |
| *B. henselae* and *D. caninum* | 7.1 | 0.0 | 0.0 | 15.4 | 0.0 | 0.0 | 0.0 | 0.0 | 0.0 | 0.0 | 0.0 | 0.0 | 0.0 |
| *B. henselae* and *M. haemofelis* | 7.1 | 0.0 | 50.0 | 0.0 | 0.0 | 0.0 | 0.0 | 0.0 | 0.0 | 0.0 | 0.0 | 0.0 | 0.0 |
| Co-infected fleas (%) | 14.3 | 0.0 | 50.0 | 15.4 | 0.0 | 0.0 | 0.0 | 0.0 | 0.0 | 0.0 | 0.0 | 0.0 | 0.0 |
| N° fleas with at least one pathogen | 28 | 1 | 4 | 13 | 0 | 0 | 0 | 0 | 0 | 0 | 3 | 2 | 5 |
|  | | | | | | | | | | | | | |

Note: Prevalences refer to flea pools (given extractions at cat individual level have been obtained of a pool of fleas) with one or more pathogens.
