# Supplementary material for: A community approach for pathogens and their arthropod vectors (ticks and fleas) in cats of sub-Saharan Africa
Source: Parasit Vectors. 2022 Sep 9;15:321. doi: 10.1186/s13071-022-05436-y (PMC9461260; doi:10.1186/s13071-022-05436-y)
Supplement: Supplementary file 2 — Additional file 2. Capture form. [file 13071_2022_5436_MOESM2_ESM.docx]

| **DCF 1** | **FIELD ASSESSMENT (Individual animal)** | (Fix barcode in this box) |
| --- | --- | --- |

Time = 24 hour clock Date format dd/mmm/yyyy

| **Date** | |  | | | | | | | | | | **Time** | | | |  | | | | | | **Animal no.** | | | |  | | | |
| --- | --- | --- | --- | --- | --- | --- | --- | --- | --- | --- | --- | --- | --- | --- | --- | --- | --- | --- | --- | --- | --- | --- | --- | --- | --- | --- | --- | --- | --- |
| **Owner consent, animal details and history:** O **Dog** O **Cat to participate in survey** | | | | | | | | | | | | | | | | | | | | | | | | | | | | | |
| **Owner :** I give mermission for my animal to participate (as per information sheet): | | | | | | | | | | | | **Signature:** | | | |  | | | | | | **Date:** | | | |  | | | |
| **Age (months)** | |  | | | | **Breed** | | | | | |  | | | | | | | | **Sex** | | O **Male** O **Female** | | | | | | | |
| **Diet** | | O Homemade O Tinned O Kibble O Mixed | | | | | | | | | | | | | | | | | | | | | | | | | | | |
| **Last deworming:** | | O Never O 1-6 months ago  O < 1 month ago O >6 months ago | | | | | | | | | | | | | | **Dewormer used:** | | | | | |  | | | | | | | |
| **Last ectoparasiticide** | | O Never O 1-6 months ago  O < 1 month ago O >6 months ago | | | | | | | | | | | | | | **Ectoparasiticide used:** | | | | | |  | | | | | | | |
| **Last vaccination** | | O Never O Within last 3 years  O Within last year | | | | | | | | | | | | | | **Vaccines used:** | | | | | | 🞏 Rabies 🞏 Leptospira  🞏 DHP 🞏 CPi/*Bordetella*  🞏 Other: __________________________ | | | | | | | |
| **Housing** | | O Indoor O Free roaming O Yard | | | | | | | | | | | | | | **Other pets?** | | | | | | O Yes O No | | | | | | | |
| **If "yes" to "Other pets": Cats or dogs and how many?** | | | | | | | | | | | | | | | | O Dog(s) __________________ O Cat(s)__________________ | | | | | | | | | | | | | |
| **Geographical details** | | | | | | | | | | | | | | | | | | | | | | | | | | | | | |
| **Pet origin (name of city/town/village)** | | |  | | | | | | | | | | | | | **GPS coordinates of sampling site** | | | | | |  | | | | | | | |
| **Physical examination and general health assessment** | | | | | | | | | | | | | | | | | | | | | | | | | | | | | |
| **Any major abnormal clinical signs evident?** | | | | | | | | | | | | O Yes O No | | | | | | | **If yes, provide details below:** | | | | | | | | | | |
|  | | | | | | | | | | | | | | | | | | | | | | | | | | | | | |
| **Body Condition Score** | | | | O 1 O 2 O 3 O 4 O 5 | | | | | | | | | | **Mucous membrane colour** | | | | | | | O Normal (pink & moist) O Pale O Icteric O Other _________________________________ | | | | | | | | |
| **Temperature (°C)** | | | |  | | | | | | **Pulse (beats/minute)** | | | | | |  | | | | | **Respiration rate (/minute)** | | | | | | |  | |
| **Ectoparasite assessments** | | | | | | | | | | | | | | | | | | | | | | | | | | | | | |
| **Ticks** | **Area 1** | | | O 1 ticks O 2 ticks O ≥ 3 ticks | | | | | | | | | | | |   1. Outside hind legs  2. Tail and anal area  3. Lateral area, not shoulders  4. Abdomen & inside hind legs  5. Shoulder and fore-legs  6. Head and neck  7. Dorsal, shoulders to tail base | | | | | | | | | | | | | |
|  | **Area 2** | | | O 1 ticks O 2 ticks O ≥ 3 ticks | | | | | | | | | | | |  |  |  |  |  |  |  |  |  |  |  |  |  |  |
|  | **Area 3** | | | O 1 ticks O 2 ticks O ≥ 3 ticks | | | | | | | | | | | |  |  |  |  |  |  |  |  |  |  |  |  |  |  |
|  | **Area 4** | | | O 1 ticks O 2 ticks O ≥ 3 ticks | | | | | | | | | | | |  |  |  |  |  |  |  |  |  |  |  |  |  |  |
|  | **Area 5** | | | O 1 ticks O 2 ticks O ≥ 3 ticks | | | | | | | | | | | |  |  |  |  |  |  |  |  |  |  |  |  |  |  |
|  | **Area 6** | | | O 1 ticks O 2 ticks O ≥ 3 ticks | | | | | | | | | | | |  |  |  |  |  |  |  |  |  |  |  |  |  |  |
|  | **Area 7** | | | O 1 ticks O 2 ticks O ≥ 3 ticks | | | | | | | | | | | |  |  |  |  |  |  |  |  |  |  |  |  |  |  |
| **Fleas** | **Overall** | | | O 1-10 fleas O 11-50 fleas O >50 fleas | | | | | | | | | | | |  |  |  |  |  |  |  |  |  |  |  |  |  |  |
| **Lice** | **Overall** | | | O 1-10 lice O 11-50 lice O >50 lice | | | | | | | | | | | |  |  |  |  |  |  |  |  |  |  |  |  |  |  |
| **Ectoparasite specimen collection (single jar with 70% ethanol per animal)** | | | | | | | | | | | | | | | | | | | | | | | | | | | | | |
| **Up to 30 ticks collected:** | | | | | O Yes O No | | | | | | | **Fleas and lice:** | | | | | | O Collected as many as possible O None present | | | | | | | | | | | |
| **Ensured that the barcode on the collection jar corresponds with barcode on this form** | | | | | | | | | | | | | | | | | | | | | | | | O Yes | | | | | |
| **Blood collection and processing and anal swab preparation** | | | | | | | | | | | | | | | | | | | | | | | | | | | | | |
| **Blood specimen collected** | | | | | O Yes O No | | | | | | **FTA card used** | | | | | | O Yes O No | | | | | | **4DxPlus kit used** | | | | O Yes O No | | |
| **4Dx Plus kit positive results:** | | | | | | | 🞏 Heartworm 🞏 *Borrelia burgdorferi* (Lyme disease) 🞏 *Anaplasma* spp. 🞏 *Ehrlichia* spp. | | | | | | | | | | | | | | | | | | | | | | |
| **Serum prepared** | | | | | O Yes O No | | | | | | **Anal swab performed** | | | | | | | | | | | | O Yes O No | | | | | | |
| **Rabies vaccine administered?** | | | | | | | | | O Yes O No | | | | | | **Ectoparasite preventive dispensed?** | | | | | | | | | | O Yes O No | | | |  |
| **Field assessment performed by:** | | | | | | | | **Signature** | | |  | | | | | | | | | | | | **Date:** |  | | | | | |
| **Preparation for shipment checklist** | | | | | | | | | | | | | | | | | | | | | | | | | | | | | |
| **Following was prepared for shipment to Clinvet:** | | | | | | | | | | | | | 🞏 DCF 1 🞏 FTA card (one half) 🞏 Ectoparasite collection jar 🞏 Anal swab | | | | | | | | | | | | | | | | |
| **Prepared for shipment by:** | | | | | | | | | **Signature** | |  | | | | | | | | | | | **Date:** | |  | | | | | |
